# Visceral fat area and body fat percentage measured by bioelectrical impedance analysis correlate with glycometabolism

**DOI:** 10.1186/s12902-022-01142-z

**Published:** 2022-09-15

**Authors:** Shuying Li, Shaoping Li, Jie Ding, Weihong Zhou

**Affiliations:** Nanjing Drum Tower Hospital, the Affiliated Hospital of Nanjing University Medical School, Nanjing, China

**Keywords:** T2DM, Pre-diabetes, Visceral fat area, Body fat percentage, Bioelectrical impedance analysis

## Abstract

**Background:**

Adiposity evaluated by body mass index (BMI) is associated with glycometabolism. The aim of the investigation was to explore the correlation of visceral fat area (VFA), body fat percentage (BFP), BMI and waist circumference (WC) with type 2 diabetes mellitus (T2DM) and pre-diabetes.

**Methods:**

A total of 18,458 participates underwent physical examination in Nanjing Drum Tower Hospital from January 2018 to April 2022 was included in this study. Data were collected retrospectively. Regression analysis was used to evaluate the relationship of VFA, BFP, WC and BMI with diabetes status, fasting blood glucose (FBG) and glycohemoglobin (HbA1c).

**Results:**

After fully adjusted for multiple covariates, VFA, BFP, WC and BMI in T2DM and pre-diabetes group exceeded compared with normal group. FBG was positively correlated with VFA, BFP, WC and BMI with βs of 2.221,0.306,0.606 and 0.175(*p* < 0.001). HbA1c was also positively correlated with the four indexes with βs of 2.645, 0.328, 0.685 and 0.255(*p* < 0.001). Subgroup analysis shown that FBG and HbA1c were positively correlated with VFA, BFP, BMI and WC in normal and pre-diabetes group (*p* < 0.001). FBG was negatively correlated with BMI in T2DM group (*p* = 0.023). In T2DM, there were non-linear relationships of HbA1c with VFA, BFP, WC and BMI with the inflection points for about 7%. Before the inflection point, HbA1c was positively correlated with obesity-related indicators, and it was reversed after the inflection point. In the individuals with excessive VFA and normal BMI, the risk for glycometabolism disorder exceed compared with normal VFA and normal BMI. Every per-standard deviation increasing in VFA, BFP, WC and BMI, the corresponding risk increasing of glycometabolism disorder was 16.4, 14.6, 22.6 and 22.2%.

**Conclusion:**

The study demonstrated that in adults with T2DM or prediabetes, the VFA, BFP, WC and BMI were higher than with normal glycometabolism. In pre-diabetes and normal population, there were positive correlations of HbA1c and FBG with obesity-related indicators. In T2DM with poor glycemic control (HbA1c > 7%), there might be a trend of fat loss. VFA could negatively affect glycometabolism independently from BMI. The optimum to evaluate the risk of glycometabolism disorder was WC.

## Introduction

As two major public health events in the twenty-first century, obesity and T2DM go hand in hand [1]. Obesity can raise the risk of developing T2DM, cause long-term multiple complications and increase mortality [2]. Pre-diabetes, which is defined as glycemic higher than normal but lower than diabetes threshold, is an intermediate state between diabetes and normal, and it is also a window for reversing the impaired glucose metabolism to normal [3]. The approaches for pre-diabetes is mainly to control weight and improve lifestyle [3]. Therefore, it is also very important to evaluate obesity status in patients with pre-diabetes.

To this day, the most frequently used index to evaluate obesity remains to be BMI [4]. Recently, researchers believe that the occurrence of T2DM may be closely related to the fat distribution [5, 6]. Moreover, the evaluation of fat distribution by body composition analysis is helpful to evaluate the relevant T2DM disease-related complications, such as sarcopenia and cardiovascular diseases [7]. VFA and BFP are important index of human body composition, which is closely related to metabolism and has important clinical significance in the evaluation of implicit obesity. In recent years, a growing stream of researches is of the opinion that using BFP and visceral fat to evaluate obesity is more meaningful than traditional index such as BMI or WC [8, 9]. For these indicators, whether the risk of glycometabolism disorder can be evaluated, and which of these indicators is best? At present, there is a lack of large-scale sample research on the characteristics of human body composition in patients with T2DM. Moreover, the correlation of body composition and pre-diabetes is not clear. Besides, as two important indicators for glycemic control, the relationship of HbA1c and FBG to fat distribution is not clear.

Computed tomography (CT), Magnetic Resonance Imaging (MRI) and Dual-emission X-ray Absorptiometry (DXA) are commonly used to evaluate VFA and BFP, but they are difficult to be widely used because of their high requirements of facilities, high expense and complex operation. In recent years, since its simple operation and low cost, bioelectrical impedance analysis (BIA) method is becoming more and more popular [10]. Bioelectrical impedance analysis (BIA) is a method to indirectly test the body fat content by measuring the body water volume, and is also the most commonly used body composition test method. In this study, the body composition was measured by BIA to explore the correlation of VFA, BFP, BMI and WC with diabetes status, so as to evaluate the relationship between body composition and glycometabolism.

## Methods

### Study population

Participates included in the study who underwent physical examination in the health management center of Nanjing Drum Tower Hospital from January 2018 to April 2022 were screened according to inclusion and excluding criteria. Inclusion criteria. 1) Age>30 years and ≤ 90 years; 2) Have complete personal holographic files, including general information (age, sex, systolic pressure (SBP), diastolic pressure (DBP), medical history), biochemical parameters (FBG, HbA1c, high-density lipoprotein cholesterol (HDL-c), low-density lipoprotein cholesterol (LDL-c), total cholesterol (TC), serum uric acid (UA), etc.), and data of body composition parameters collected through bioelectric impedance analysis. Excluding criteria: 1) refuse to answer medical history; 2) diagnosed with type 1 diabetes; 3) pregnant; 4) taking steroids such as glucocorticoids; 5) Unreasonable data (e.g.WC of 0 cm) after data quality control.

### Data collection

General information of participates, such as gender, age, medical history (including hypertension (HBP), diabetes, medication history, etc.) was retrospective collected. Body parameters, BIA results and serum biochemical indexes were retrospective collected. All tests were performed on empty stomach individuals in the morning. The sequence of different tests were allocated according to the call system of the health management center.

Body parameters were measured by trained researchers. Blood pressure measurement: let the subject calm down in a quiet environment for 10–20 minutes, and then use an electronic sphygmomanometer to measure blood pressure. All subjects on an empty stomach relieve oneself, remove metal ornaments, and wear light clothes to measure their height, weight, waist circumference, and body composition in the morning. Use height and electronic scale to measure height and weight. WC was measured by a soft ruler.

Use h-key350 human body composition analyzer (Beijing Sihai Huachen Technology Co., Ltd.) to measure the VFA of human body. During the measurement, the individuals stood on the instrument barefoot to ensure that both feet were in full contact with the foot electrode position of human body composition analyzer. Hold the handles on both sides with both hands and ensure that the five fingers are in full contact with the test electrode. Straighten arms to both sides and maintain an included angle of about 15 degrees with torso. Entered personal information such as age, gender, and height for correction. Clicked to start the detection, waited about 2 min, and then the BFP and VFA results were automatically transmitted to the connected computer for storage and archiving. The test requirements are as follows: (1) vigorous exercise or other physical activities are not recommended within 2 hours before the test; (2) Bathing within 2 hours before the test is not recommended; (3) The indoor temperature should be kept between 21 and 25 °C; (4) It is recommended to use the toilet before the test, because human excreta will temporarily change the body composition.

Serum biochemical indexes: take 3 ml of peripheral venous blood under the fasting state of the subject, and use the automatic biochemical analyzer to detect FBG, TC, HDL-c, LDL-c, TG, etc.

### Ethic

The research is based on the project “ construction of life cycle intelligent monitoring and management service system based on DRGs” approved by the Ethics Committee of Nanjing Drum Tower Hospital(2022–046-01).

### Statistical analysis

Continuous variables such as age, SBP and DBP were expressed in the form of mean ± standard deviation (SD), and classified variables such as sex, DM and HBP were expressed in the form of percentage(%). The diagnosis of diabetes came from previous history and/or FBG ≥ 7.0mmo/l and/or HbA1c ≥ 6.5% [11]. The diagnosis of normal glycometabolism should meet the following three conditions: 1) no previous history of diabetes; 2) FBG < 5.5 mmol/; 3) HbA1c < 5.7%. Excluding the participation system of diabetes and normal blood glucose metabolism, the others were pre-diabetes [12]. The diagnosis of HBP came from previous history and/or SBP ≥ 140 mmHg and/or DBP ≥ 90 mmHg. BMI ≥ 28 kg/m^2^ was defined as BMI obesity. VFA ≥ 100 cm^2^ was VFA obesity. BFP>25% for males or>35% for females was defined as BFP obesity [11, 13]. WC ≥ 90 cm for males or ≥ 80 cm for females was defined as WC obesity [14]. Regression analysis of VFA, BFP, BMI and WC among participants with different diabetes status was conducted. At the same time, the relationship of HbA1c and FBG with VFA, BFP, BMI and WC explore. The relationship of HbA1c with VFA, BFP, WC and BMI in patients with T2DM was explore by smooth fit curve and threshold effect analysis. All analyses were performed with Empower Stats (http://www.empowerstats.com). *P* < 0.05 indicated the difference of statistically significant.

## Results

### Comparison of baseline information with different diabetes status

A total of 18,458 participants were included in the study. According to the different diabetes status, the population was divided into three groups (9759 cases with normal glycometabolism, 6698 cases with pre-diabetes and 2001 cases T2DM). VFA in normal group, pre-diabetes group and T2DM group were 84.546 ± 30.795, 93.614 ± 33.489, 99.311 ± 36.381 cm^2^ (*p* < 0.001), respectively. BFP were 27.591 ± 6.449, 29.019 ± 6.687, 28.996 ± 6.898 (%) (*p* < 0.001), respectively. WC were 86.362 ± 9.478, 88.813 ± 9.805, 91.327 ± 10.116 cm (*p* < 0.001), respectively. BMI were 23.997 ± 3.145, 24.902 ± 3.231, 25.810 ± 3.360 kg/m^2^ (*p* < 0.001), respectively. See Table 1 for details.

**Table 1:** Baseline characteristics of individuals included in the study grouped by diabetes status

Items	Normal (*n* = 9759)	Pre-Diabetes (*n* = 6698)	T2DM (*n* = 2001)	*P*-value
Age (years)	48.054 ± 9.774	53.434 ± 9.407	56.187 ± 8.902	< 0.001
Sex = male(%)	52.751	54.673	69.015	< 0.001
HBP(%)	31.796	48.104	64.168	< 0.001
SUA (μmol/L)	343.421 ± 92.982	361.539 ± 94.726	353.431 ± 92.257	< 0.001
TC (mmol/L)	4.932 ± 0.918	5.153 ± 0.981	5.029 ± 1.128	< 0.001
HDL-c (mmol/L)	1.402 ± 0.385	1.354 ± 0.372	1.226 ± 0.331	< 0.001
LDL-c (mmol/L)	2.876 ± 0.758	3.072 ± 0.815	2.943 ± 0.902	< 0.001
TG (mmol/L)	1.401 ± 1.119	1.635 ± 1.314	2.058 ± 1.834	< 0.001
FBG (mmol/L)	4.744 ± 0.368	5.232 ± 0.587	7.787 ± 2.493	< 0.001
HbA1c(%)	5.295 ± 0.248	5.825 ± 0.274	7.449 ± 1.484	< 0.001
VFA (cm2)	84.546 ± 30.795	93.614 ± 33.489	99.311 ± 36.381	< 0.001
BFP (%)	27.591 ± 6.449	29.019 ± 6.687	28.996 ± 6.898	< 0.001
WC (cm)	86.362 ± 9.478	88.813 ± 9.805	91.327 ± 10.116	< 0.001
BMI (kg/m2)	23.997 ± 3.145	24.902 ± 3.231	25.810 ± 3.360	< 0.001

Mean ± SD for continuous variables, *p*-value was calculated by analysis of variance. % for categorical variables, *p*-value was calculated by chi-square test. *BMI* Body mass index, *SUA* Serum uric acid, *HbA1c* Glycosylated hemoglobin, *FBG* Fasting blood glucose, *HBP* High blood pressure, *HDL-c* High density lipoprotein cholesterol, *LDL-c* Low density lipoprotein cholesterol, *TC* Total cholesterol, *TG* Triglyceride, *VFA* Visceral fat area, *BFP* Body fat percentage, *WC* Waist circumference

### Relationship between adiposity and glycometabolism

The group with normal glycometabolism was taken as the reference. After fully adjusting for multiple covariates (sex, age, HBP, UA, TG, TC, HDL-C and LDL-C), the VFA of pre-diabetes group and T2DM group were 3.466 and 8.259 cm higher than normal group (*p* < 0.001), respectively. The BFP was 0.446 and 0.998% higher than normal (*p* < 0.001). The WC was 1.253 and 2.340 cm higher (*p* < 0.001), and the BMI was 0.410 and 0.853 kg/m2 higher than normal group (*p* < 0.001). Subgroup analysis showed that the difference between groups still existed. See Table 2 for details.

**Table 2:** Relational ship between diabetes status and obesity

	β (95% CI) *p*-value			
	VFA (cm2)	BFP(%)	WC (cm)	BMI (kg/m^2^)
Normal	reference	reference	reference	reference
Pre-Diabetes				
Total	3.466 (2.512, 4.420) < 0.001	0.446 (0.280, 0.613) < 0.001	1.253 (0.989, 1.517) < 0.001	0.410 (0.319, 0.500) < 0.001
Females	3.373 (1.843, 4.904) < 0.001	0.425 (0.166, 0.683) 0.001	1.097 (0.727, 1.466) < 0.001	0.347 (0.211, 0.484) < 0.001
Males	3.223 (2.031, 4.415) < 0.001	0.426 (0.210, 0.642) < 0.001	1.277 (0.909, 1.645) < 0.001	0.426 (0.305, 0.546) < 0.001
T2DM				
Total	8.259 (6.769, 9.749) < 0.001	0.988 (0.728, 1.248) < 0.001	2.340 (1.928, 2.753) < 0.001	0.853 (0.711, 0.995) < 0.001
Females	10.422 (7.639, 13.205) < 0.001	1.246 (0.776, 1.716) < 0.001	2.736 (2.064, 3.408) < 0.001	1.151 (0.903, 1.400) < 0.001
Males	6.917 (5.212, 8.622) < 0.001	0.814 (0.505, 1.122) < 0.001	2.098 (1.570, 2.625) < 0.001	0.695 (0.523, 0.867) < 0.001

Age, sex, HBP, TG, TC, LDL-c, HDL-c and SUA were adjusted. Sex was not adjusted for in the subgroup analysis. *SUA* Serum uric acid, *HbA1c* Glycosylated hemoglobin, *FBG* Fasting blood glucose, *HBP* High blood pressure, *HDL-c* High density lipoprotein cholesterol, *LDL-c* Low density lipoprotein cholesterol, *TC* Total cholesterol, *TG* Triglyceride, *BMI* Body mass index, *VFA* Visceral fat area, *BFP* Body fat percentage, *WC* Waist circumference, *T2DM* Type 2 diabetes mellitus

After fully adjusting multiple covariates, VFA, BFP, WC, BMI were positively correlated with FBG(β (95% CI) *p*-value = 2.221 (1.879, 2.564) < 0.001, 0.306 (0.246, 0.365) < 0.001, 0.606 (0.511, 0.700) < 0.001 and 0.175 (0.143, 0.208) < 0.001, respectively). After subgroup analysis, this correlation still existed in the normal group and the pre-diabetes group. But in T2DM group, VFA, BFP and WC had no correlation with FBG. BMI was negatively correlated with FBG. After fully adjustment for multiple covariate, VFA, BFP, WC, BMI were positively correlated with HbA1c(β (95% CI) *p*-value = 2.645 (2.119, 3.172) < 0.001, 0.328 (0.236, 0.420) < 0.001, 0.685 (0.539, 0.831) < 0.001 and 0.255 (0.205, 0.305) < 0.001, respectively). After subgroup analysis, this correlation still existed in the normal group and the pre-diabetes group. But in the T2DM group, VFA, BFP, WC and BMI had no correlation with HbA1c. See Tables 3 and 4 for details.

**Table 3:** Relationship between FBG and obesity

	β (95% CI) *p*-value			
	VFA (cm2)	BFP(%)	WC (cm)	BMI (kg/m^2^)
Total	2.221 (1.879, 2.564) < 0.001	0.306 (0.246, 0.365) < 0.001	0.606 (0.511, 0.700) < 0.001	0.175 (0.143, 0.208) < 0.001
Normal	8.983 (7.454, 10.511) < 0.001	1.476 (1.197, 1.755) < 0.001	2.872 (2.446, 3.297) < 0.001	0.800 (0.653, 0.948) < 0.001
Pre-Diabetes	6.112 (4.848, 7.376) < 0.001	0.880 (0.664, 1.095) < 0.001	2.019 (1.675, 2.363) < 0.001	0.552 (0.433, 0.670) < 0.001
T2DM	0.380 (−0.245, 1.005) 0.234	0.072 (−0.028, 0.172) 0.157	− 0.055 (− 0.228, 0.118) 0.531	−0.068 (− 0.127, − 0.009) 0.023

Age, sex, HBP, TG, TC, LDL-c, HDL-c and SUA were adjusted. *SUA* Serum uric acid, *HbA1c* Glycosylated hemoglobin, *FBG* Fasting blood glucose, *HBP* High blood pressure, *HDL-c* High density lipoprotein cholesterol, *LDL-c* Low density lipoprotein cholesterol, *TC* Total cholesterol, *TG* Triglyceride, *BMI* Body mass index, *VFA* Visceral fat area, *BFP* Body fat percentage, *WC* Waist circumference, *T2DM* Type 2 diabetes mellitus

**Table 4:** Relationship between HbA1c and obesity

	β (95% CI) *p*-value			
	VFA (cm2)	BFP(%)	WC (cm)	BMI (kg/m^2^)
Total	2.645 (2.119, 3.172) < 0.001	0.328 (0.236, 0.420) < 0.001	0.685 (0.539, 0.831) < 0.001	0.255 (0.205, 0.305) < 0.001
Normal	4.597 (2.329, 6.865) < 0.001	0.526 (0.113, 0.939) 0.013	1.639 (1.007, 2.272) < 0.001	0.654 (0.436, 0.873) < 0.001
Pre-Diabetes	4.755 (2.063, 7.447) < 0.001	0.633 (0.175, 1.091) 0.007	1.467 (0.732, 2.202) < 0.001	0.664 (0.412, 0.917) < 0.001
T2DM	0.220 (−0.813, 1.253) 0.676	0.062 (−0.103, 0.226) 0.462	− 0.218 (− 0.504, 0.067) 0.134	−0.075 (− 0.173, 0.022) 0.128

Age, sex, HBP, TG, TC, LDL-c, HDL-c and SUA were adjusted. *SUA* Serum uric acid, *HbA1c* Glycosylated hemoglobin, *FBG* Fasting blood glucose, *HBP* High blood pressure, *HDL-c* High density lipoprotein cholesterol, *LDL-c* Low density lipoprotein cholesterol, *TC* Total cholesterol, *TG* Triglyceride, *BMI* Body mass index, *VFA* Visceral fat area, *BFP* Body fat percentage, *WC* Waist circumference, *T2DM* Type 2 diabetes mellitus

### Nonlinear relationship between adiposity and HbA1c in T2DM

**Fig. 1:**
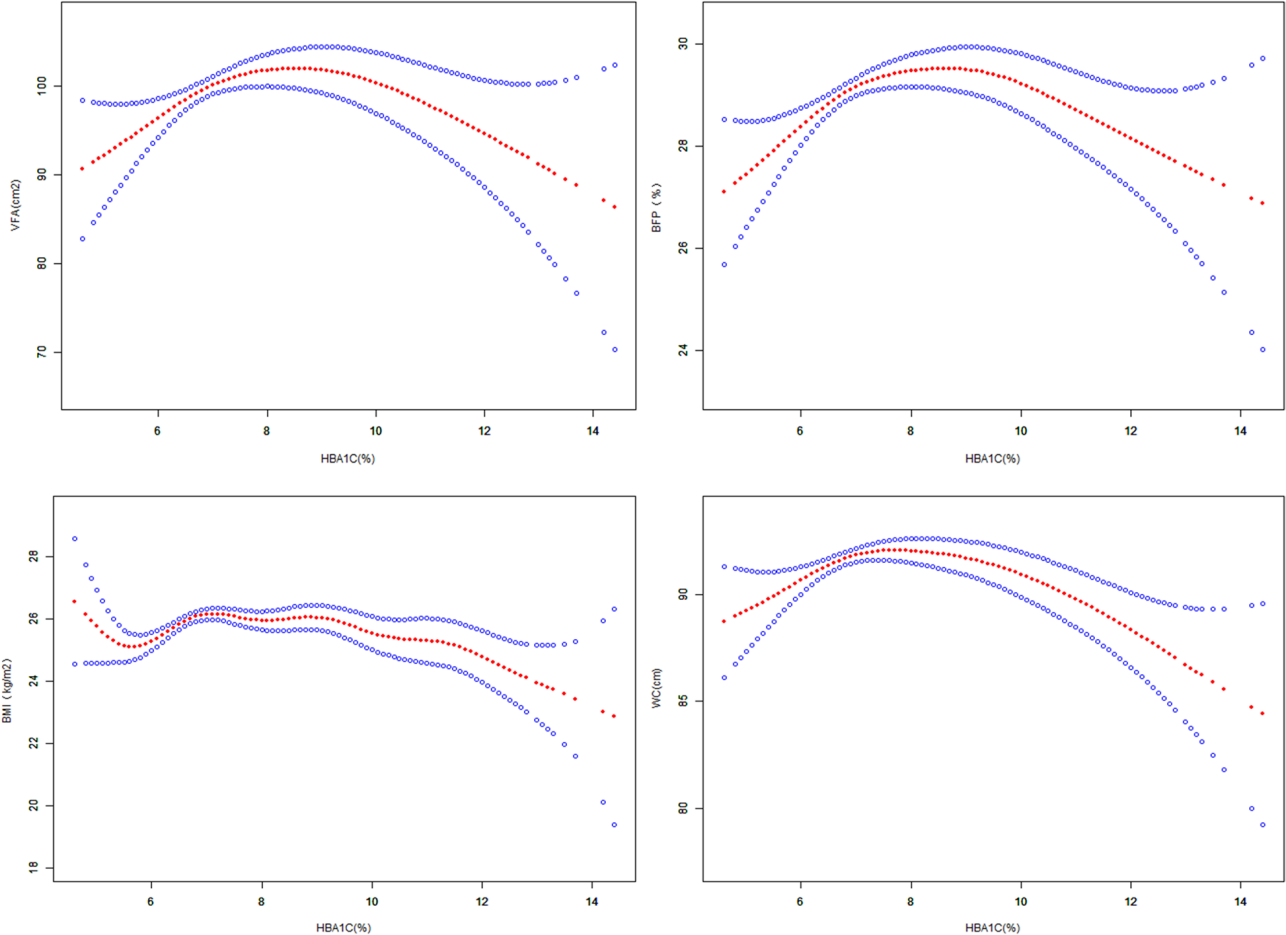
Non-liner relationship between obesity and HBA1c in T2DM. Age, sex, HBP, TG, TC, LDL-c, HDL-c and SUA were adjusted. SUA: serum uric acid. HbA1c: Glycosylated hemoglobin. FBG: Fasting blood glucose. HBP: High blood pressure. HDL-c: High density lipoprotein cholesterol. LDL-c: Low density lipoprotein cholesterol. TC: Total cholesterol. TG: Triglyceride. BMI: body mass index. VFA: Visceral fat area. BFP: Body fat percentage. WC: Waist circumference. T2DM: Type 2 diabetes mellitus

Smooth fitting curve and threshold effect analysis explored the nonlinear relationship between HbA1c and VFA, BFP, WC and BMI. It was found that VFA, BFP, WC and BMI showed an inverted U-shaped curve with the change of HbA1c. VFA, BFP, BMI and WC had threshold effect with the change of HbA1c, with the inflection points of 7, 6.7, 7 and 7% respectively. See Table 5 and Fig. 1 for details.

**Table 5:** Threshold effect analysis of HbA1c and obesity in T2DM

Outcome	VFA (cm2)	BFP(%)	WC (cm)	BMI (kg/m^2^)
Fitting by standard linear model	0.220 (−0.813, 1.253) 0.676	0.062 (−0.103, 0.226) 0.462	− 0.218 (− 0.504, 0.067) 0.134	−0.075 (− 0.173, 0.022) 0.128
Fitting by two-piecewise linear model Inflection point	7	6.7	7	7
<Inflection point	7.471 (3.565, 11.377) < 0.001	1.799 (0.998, 2.601) < 0.001	2.238 (1.160, 3.316) < 0.001	0.810 (0.443, 1.176) < 0.001
> Inflection point	−1.292 (−2.586, 0.003) 0.051	−0.146 (− 0.335, 0.043) 0.129	−0.730 (− 1.088, − 0.373) < 0.001	−0.260 (− 0.381, − 0.139) < 0.001
Log-likelihood ratio	< 0.001	< 0.001	< 0.001	< 0.001

Age, sex, HBP, TG, TC, LDL-c, HDL-c and SUA were adjusted. *SUA* Serum uric acid, *HbA1c* Glycosylated hemoglobin, *FBG* Fasting blood glucose, *HBP* High blood pressure, *HDL-c* High density lipoprotein cholesterol, *LDL-c* Low density lipoprotein cholesterol, *TC* Total cholesterol, *TG* Triglyceride, *BMI* Body mass index, *VFA* Visceral fat area, *BFP* Body fat percentage, *WC* Waist circumference

### Comparison of different adiposity indexes to evaluate the risk of glycometabolism disorder

According to different obesity status for the indexes, the participants were divided into 4 groups. Group 1: normal VFA/BFP/WC and normal BMI. Group 2: VFA/BFP/WC obesity and normal BMI. Group 3: normal VFA/BFP/WC and BMI obesity. Group 4: VFA/BFP/WC obesity and BMI obesity. Taking group 1 as the reference group, explore the risk of glycometabolism disorder (pre-diabetes and T2DM) in groups 2 to 4. The study found that in group 2, the risk of glycometabolism disorder in the VFA obesity and BMI normal group was higher than that in group 1. However, there was no significant difference in the risk of glycometabolism disorder for the groups with BFP or WC obesity and normal BMI compared with group 1. See Table 6 for details.

**Table 6:** Risk for glycometabolism disorder (T2DM and pre-diabetes) in different group classified by VFA/BFP/WC and BMI

Groups	Risk for glycometabolism disorder (OR (95% CI) *p*-value)		
	Grouped by VFA and BMI	Grouped by BFP and BMI	Grouped by WC and BMI
1	reference	reference	reference
2	1.615 (1.267, 2.058) < 0.001	1.248 (0.909, 1.712) 0.170	1.025 (0.376, 2.797) 0.961
3	1.229 (1.130, 1.338) < 0.001	1.160 (1.078, 1.248) < 0.001	1.256 (1.168, 1.349) < 0.001
4	1.617 (1.460, 1.790) < 0.001	1.674 (1.511, 1.855) < 0.001	1.764 (1.590, 1.958) < 0.001

Group 1:non-excessive BMI and non-excessive VFA/BFP/WC; Group 2: non-excessive BMI and VFA/BFP/WC obesity; Group 3: non-excessive VFA/BFP/WC and BMI obesity. Group 4: BMI obesity and VFA/BFP/WC obesity. Normal = 0, T2DM and Prediabetes = 1. Age, sex, HBP, TG, TC, LDL-c, HDL-c and SUA were adjusted. *SUA* Serum uric acid, *HbA1c* Glycosylated hemoglobin, *FBG* Fasting blood glucose, *HBP* High blood pressure, *HDL-c* High density lipoprotein cholesterol, *LDL-c* Low density lipoprotein cholesterol, *TC* Total cholesterol, *TG* Triglyceride, *BMI* Body mass index, *VFA* Visceral fat area, *BFP* Body fat percentage, *WC* Waist circumference, *T2DM* Type 2 diabetes mellitus

After fully adjusting multiple covariates, every 1 cm^2^ increase in VFA corresponded for 0.005 increasing in the risk of glycometabolism disorder (β(95% CI) *p*-value = 1.005 (1.004, 1.006) < 0.001). For every 1% increase in BFP, the risk of glycometabolism disorder increased by 0.022(β(95% CI) *p*-value =1.022 (1.016, 1.029) < 0.001). For every 1 cm increase in WC, the risk of glycometabolism disorder increased by 0.023(β(95% CI) *p*-value =1.023 (1.019, 1.027) < 0.001). For every 1 kg/m2 increase in BMI, the risk of glycometabolism disorder increased by 0.068(β (95% CI) *p*-value =1.068 (1.056, 1.080) < 0.001). For per standard deviation increasing in VFA, BFP, WC and BMI, the risk of glycometabolism disorder increased by 16.4, 14.6, 22.6 and 22.2%. See details in Table 7.

**Table 7:** VFA/BFP/WC/BMI and the risk for glycometabolism disorder

Index	SD	β (95% CI) *p*-value	Risk increase for glucose metabolism disorder in every 1 SD increase
VFA (cm2)	32.883	1.005 (1.004, 1.006) < 0.001	0.164
BFP (%)	6.624	1.022 (1.016, 1.029) < 0.001	0.146
WC (cm)	9.812	1.023 (1.019, 1.027) < 0.001	0.226
BMI (kg/m2)	3.259	1.068 (1.056, 1.080) < 0.001	0.222

Age, sex, HBP, TG, TC, LDL-c, HDL-c and SUA were adjusted. β indicate the correlation of glycometabolism disorder with VFA, BFP, WC and BMI(T2DM and Pre-diabetes = 1, normal = 0). *SUA* Serum uric acid, *HbA1c* Glycosylated hemoglobin, *FBG* Fasting blood glucose, *HBP* High blood pressure, *HDL-c* High density lipoprotein cholesterol, *LDL-c* Low density lipoprotein cholesterol, *TC* Total cholesterol, *TG* Triglyceride, *BMI* Body mass index, *VFA* Visceral fat area, *BFP* Body fat percentage, *WC* Waist circumference, *T2DM* Type 2 diabetes mellitus

## Discussion

Adiposity is closely related to glycometabolism. In addition to BMI, our study indicated that VFA, BFP, WC were associated with glycometabolism disorder. Individuals with glycometabolism disorder had higher BFP, VFA, WC and BMI compared with those with normal glycometabolism. FBG and HbA1c were positively correlated with VFA, BFP, WC and BMI, respectively. In patients with pre-diabetes or normal individuals, there is a positively correlation between fat distribution and deterioration of glycemic control. However in individuals with T2DM, the correlations disappeared or reversed. In T2DM, the association of HbA1c and VFA, BFP, WC and BMI showed a nonlinear inverted U shape with an inflection about 7%. Before 7%, there is a positive correlation, and after 7%, it was opposite, suggesting that patients with poorly controlled T2DM may be with a trend of fat loss. VFA independent from BMI could negatively affect glycometabolism. WC might be optimum to evaluate the risk of glycometabolism disorder.

As we all know, adiposity is an independent risk factor for glycometabolism disorder since the accumulation of fat can affect the effects of insulin through a variety of mechanisms [15, 16]. Pancreatic β cell dysfunction and insulin resistance in multiple organs (such as liver and muscle) could be caused by obesity [2, 15]. In recent years, the incidence of T2DM has been increasing in the worldwide, which may be related to obesity caused by poor diet habits. As the traditional index for obesity, BMI is linearly positively correlated with the risk of T2DM [15]. However, BMI is limited. For example, BMI cannot reflect the effect of fat distribution on glycometabolism [17]. Therefore, a growing number of researches is exploring the relationship between body composition and glycometabolism. At present, magnetic resonance imaging, computed tomography and dual energy X-ray for body composition measurement cannot been widely used because of high cost and high demanding venue [8, 9]. BIA can compensate for the shortcomings above [18]. This study explored the relationship between adiposity related indicators measured by BIA and glycometabolism disorders.

After fully adjusting for multiple variables, VFA of individuals with pre-diabetes and T2DM were 3.5cm^2^ and 8.3cm^2^ higher than those with normal glycometabolism, BFP was 0.4 and 1.0% higher, WC was 1.3 cm and 2.3 cm higher, and BMI was 0.4 kg/m^2^ and 0.9 kg/m^2^ higher, respectively. HbA1c and FBG were positively correlated with VFA, BFP, WC and BMI. This suggests that obesity-related indexes measured by BIA are consistent with BMI in judging the risk of glycometabolism disorders. It was found that the incidence of T2DM was positively correlated with VFA [19]. Among individuals with normal glycometabolism and pre-diabetes, VFA, BFP, WC and BMI were positively correlated with HbA1c, but not in T2DM. A previous study found that HbA1c had no clear correlation with BMI and VFA in T2DM, while glycated albumin (GA) and GA/HbA1c had a negative correlation [20]. The association of HbA1c and VFA, BFP, WC and BMI showed a nonlinear inverted U shape with an inflection about 7% in T2DM. Before 7%, there is a positive correlation, and after 7%, it seemed to be opposite. HbA1c reflects the glycaemic status in the latest 2–3 months, with the optimal target of lower than 7% for T2DM. Therefore, we conclude that for patients with poor glycaemic status control, there might be a trend of fat loss. It was revealed by WC and BMI. But for VFA and BFP, it was indeterminacy. Among the group with HbA1c > 7%, some individuals are T2DM diagnosis previously with poor glycemic status, and the others are newly-diagnosed T2DM. In patients of T2DM with glycaemic control, the consumption of fat increases, and the patients show a trend of emaciation, which might be the reason for the nonlinear relationship. In addition, some studies have found that diabetes is related to sarcopenia [21]. The negative correlations in BFP and VFA were not revealed maybe due to insufficient sample size or whether sarcopenia was an important source of weight loss. Besides, some studies have found that the longer the duration of T2DM, the more fat loss had [22]. Therefore, more researches are needed to demonstrate the conclusions. Our study found that visceral fat influence glycometabolism disorder independently from BMI, while other indexes are not (BFP, WC). Considering the role of visceral fat in glucose metabolism, we confirm this conclusion [23, 24]. A study believed that, compared with BMI, the accumulation of visceral fat was more valuable in predicting the occurrence of DM [25]. The visceral fat and risk of metabolic diseases in Asians are higher than that in Caucasians for the same BMI [24, 26]. This may be due to the fact that visceral fat can secrete a variety of adipokines, which have an impact on islet and insulin resistance of organs [27]. This suggests that even if BMI is not excessive, we should also pay attention to the evaluation of VFA, so as to reduce the risk of glycometabolism disorder. Every per- standard deviation increasing in WC and BMI shown higher increasing for 22.6 and 22.2% in the risk of glycometabolism disorder comparing with BFP and VF. Therefore, the status of BMI and WC as indicators for evaluating obesity cannot be completely replaced.

The study demonstrated the correlation between adiposity related indicators measured by BIA and glycometabolism disorder. Our research has many advantages: 1) our study is a rare large sample population study for evaluating glycometabolism and body composition using BIA in Asia. 2) Studies have confirmed that the fat distribution in the pre-diabetes population is related to the deterioration of blood glucose control, which reminded the pre-diabetes patients to pay more attention to the fat distribution. 3) This study confirms that VFA can affect glycometabolism independently of BMI, and reminded clinicians that they should pay attention to using BIA to evaluate implicit obesity and improve glycemic control. However there were some limitation in this study: 1) Firstly, the cross-sectional research cannot confirm the causal relationship between visceral fat accumulation and glycometabolism disorder. 2) Secondly, the study explored the relationship between adiposity and HbA1c in T2DM, but lacked of data about the duration of diabetes and lean muscle mass.3) Lifestyle such as diet and exercise, was not involved in the study, which also limit the application of our conclusion.

In conclusion, VFA, BFP, WC and BMI were positively correlated with the risk of glycometabolism disorder. Individuals of T2DM with poor glycaemic control seem to have a trend of fat loss. Even if BMI does not exceed, attention should be paid to VFA. BMI and WC cannot be completely replaced by VFA and BFP for evaluating the risk of diabetes and pre-diabetes.

## Data Availability

Data used to support the findings of this study are included within the article.
